# Primary phosphaturic mesenchymal tumour of the lumbar spine: utility of ^68^Ga-DOTATOC PET/CT findings

**DOI:** 10.1259/bjrcr.20150497

**Published:** 2016-11-02

**Authors:** Junki Maehara, Koji Yamashita, Akio Hiwatashi, Osamu Togao, Kazufumi Kikuchi, Yoshihiro Matsumoto, Kunio Iura, Yoshinao Oda, Isao Ichino, Yuji Nakamoto, Hiroshi Honda

**Affiliations:** ^1^Department of Clinical Radiology, Graduate School of Medical Sciences, Kyushu University, Fukuoka, Japan; ^2^Department of Orthopaedic Surgery, Graduate School of Medical Sciences, Kyushu University, Fukuoka, Japan; ^3^Department of Anatomic Pathology, Graduate School of Medical Sciences, Kyushu University, Fukuoka, Japan; ^4^Department of Internal Medicine, Saiseikai Fukuoka General Hospital, Fukuoka, Japan; ^5^Department of Diagnostic Imaging and Nuclear Medicine, Kyoto University Graduate School of Medicine, Kyoto, Japan

## Abstract

Primary phosphaturic mesenchymal tumours (PMTs) frequently occur in the soft tissue or bone, but rarely in the spine. The majority of patients experience long-term ostalgia and recurrent fractures. Detection of PMT can be challenging, but the clinical symptoms dramatically improve after removal of the tumour.Wepresent a case of primary PMT in the lumbar spine. CT scan showed a low-density tumour with a well-defined sclerotic margin in the L5 vertebra. MRI revealed a hypointense tumour on T_2_ weighted imaging and heterogeneous enhancement. ^68^Ga-labelled 1,4,7,10-tetraazacyclododecane-N,N′,N″, N‴-tetraacetic acid-D-Phe1-Tyr3-octreotide (^68^Ga-DOTATOC) positron emission tomography/CT scan demonstrated intense focal uptake within the tumour. Histologically, proliferation of oval to short spindle-shaped cells with fibrocollagenous stroma, abundant various-sized vessels, microcysts and thickened anastomosed bone trabeculae were seen. Mitotic figures were rarely seen. Immunohistochemically, the tumour cells were focally positive for fibroblast growth factor 23. The imaging findings in the current case are in accordance with the histological features. Among them, ^68^Ga-DOTATOC positron emission tomography/CT scan for somatostatin receptor imaging provides valuable information for determining PMT localization and characterization.

## Summary

Intraosseous primary phosphaturic mesenchymal tumours (PMT) are rare tumours that are associated with oncogenic osteomalacia.^1^ Most cases of PMT are histologically benign. However, the vast majority of patients experience symptomatic osteomalacia representing long-term ostalgia and recurrent fractures with increased fibroblast growth factor (FGF) 23 secretion. Therefore, diagnostic imaging plays an important role in early detection and subsequent treatment. Imaging findings of PMT are non-specific, leading to difficulties in making a radiological diagnosis.^2^ In this report, we present a case of PMT of the lumbar spine. CT scan, MRI and ^68^Ga-labelled 1,4,7,10-tetraazacyclododecane-N,N^′^,N^″^,N^‴^-tetraacetic acid-d-Phe^1^-Tyr^3^-octreotide (^68^Ga-DOTATOC) positron emission tomography (PET)/CT scan were performed. The resulting imaging features corresponded well with the serological and pathological characteristics of the tumour.

## Case report

A 54-year-old male had been experiencing chronic pain in his chest, back and both legs for 3 years. He was found to have hypophosphataemia and a high serum alkaline phosphatase level, and was referred to our hospital for further examination and treatment. Laboratory tests showed low serum phosphorus (2.0 mg dl^−1^), elevated serum alkaline phosphatase (933 IU l^−1^) and FGF23 (96.3 pg ml^−1^), and high urinary phosphorus (1.8 g day^−1^) levels. Based on these findings, tumour-induced osteomalacia such as PMT, which is associated with FGF23 secretion, was suspected. Systemic venous sampling for FGF23 analysis was performed. However, tumour localization was not successful.

**Figure 1.**
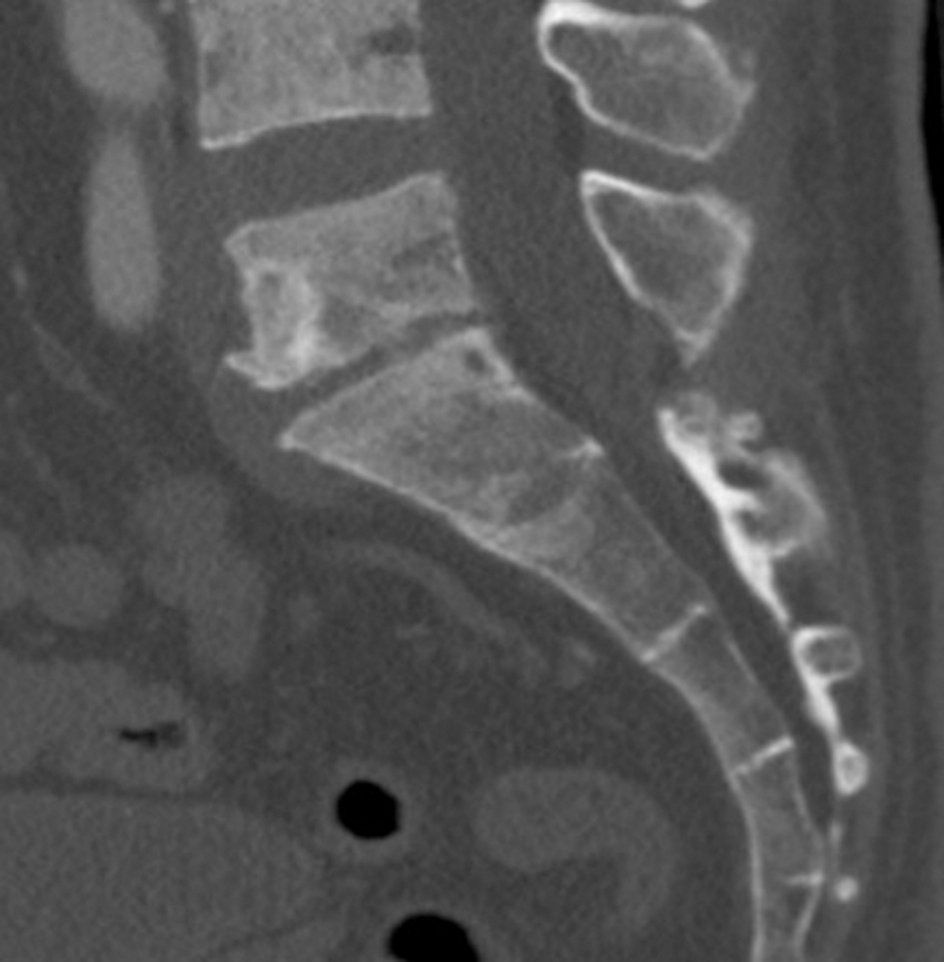
A sagittal CT image of the lumbar spine. The low-density tumour with a well-defined sclerotic margin involves the anterior aspect of the L5 vertebra.

**Figure 2.**
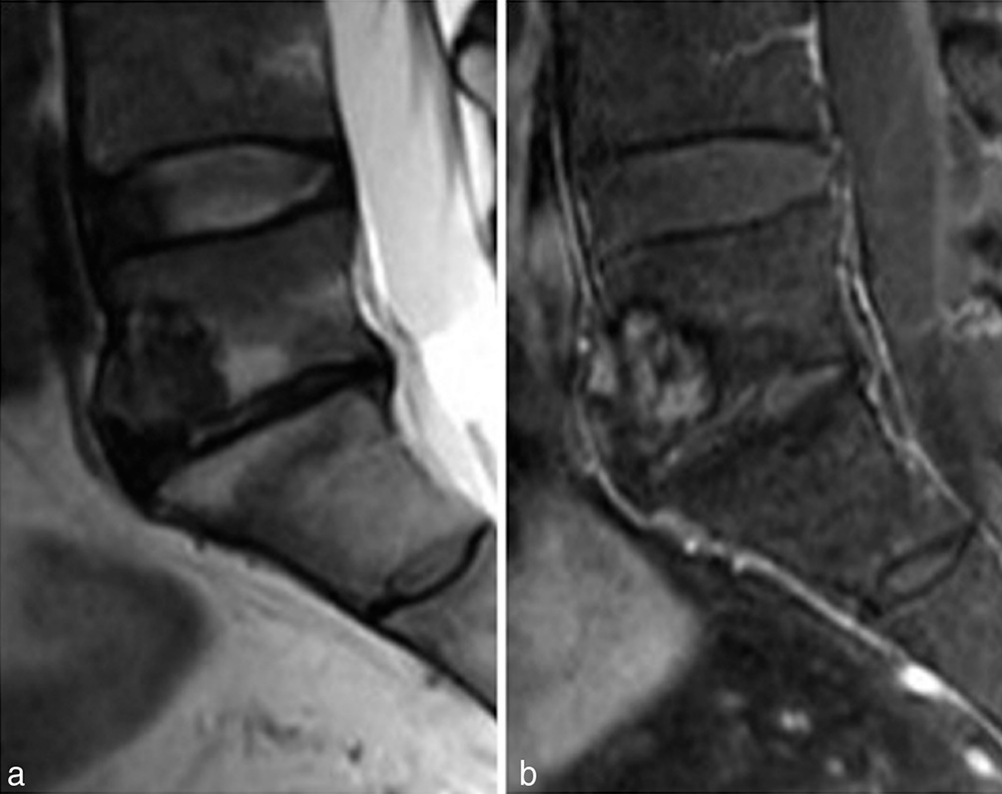
MRI of the lumbar spine. Pre-contrast *T*_2_ weighted images (a) reveal decreased signal intensity compared with the L5 vertebral body tumour. The tumour shows heterogeneous enhancement (b).

**Figure 3.**
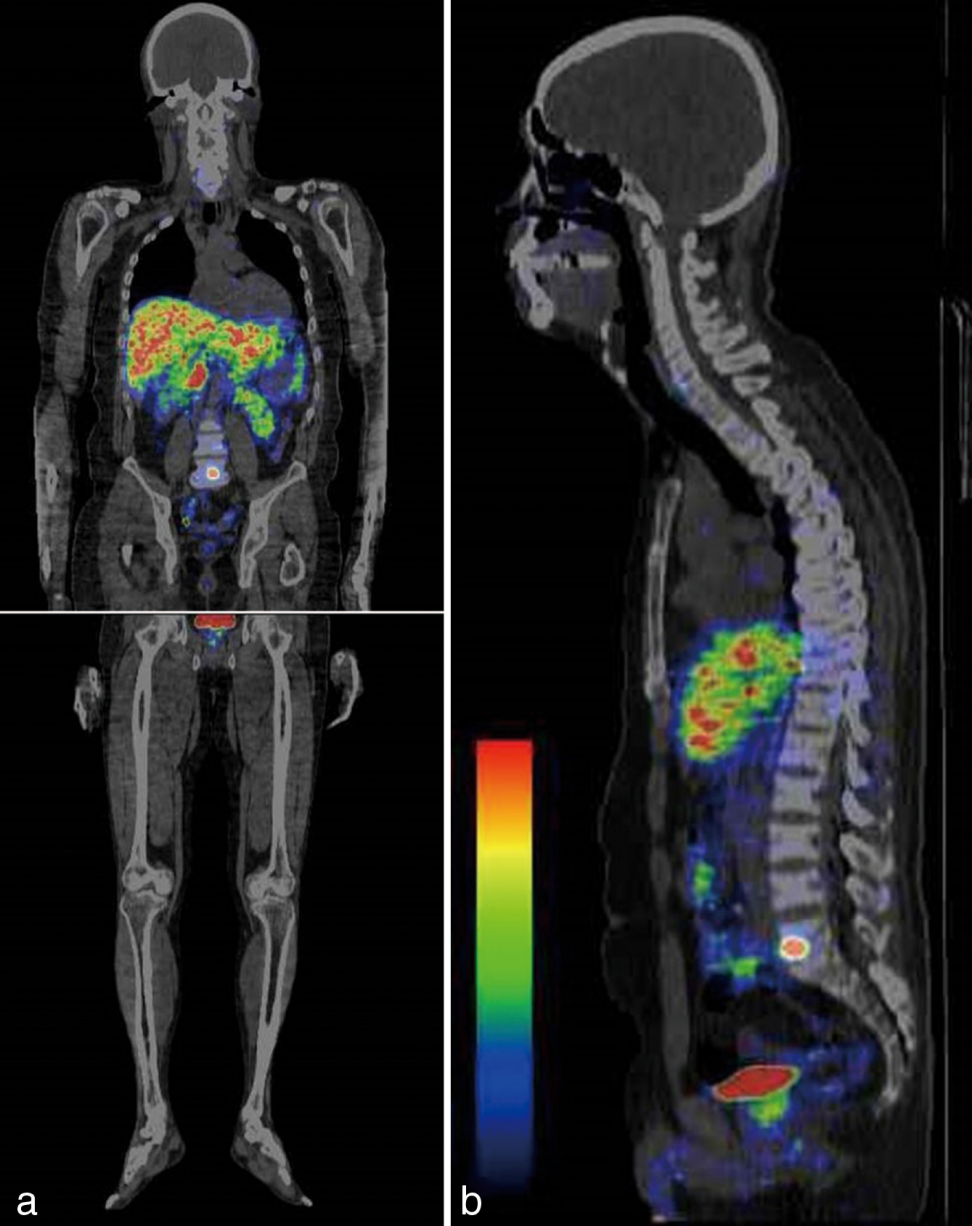
^68^Ga-DOTATOC positron emission tomography/CT scan (a, b) of the L5 vertebra demonstrates intense focal uptake within the tumour (maximum standardized uptake value = 10.5). ^68^Ga-DOTATOC positron emission tomography/CT scan did not show any abnormality in other regions.

CT scan showed a low-density tumour with a well-defined sclerotic margin in the anterior aspect of the L5 vertebra (Figure 1). On MRI, pre-contrast *T*_1_ and *T*_2_ weighted images revealed decreased signal intensity compared with the vertebral body. The tumour showed heterogeneous enhancement (Figure 2). For ^68^Ga-DOTATOC PET/CT scan, 108.3 MBq of ^68^Ga-DOTATOC was injected intravenously and whole-body PET/CT scan was performed. The ^68^Ga-DOTATOC PET/CT scan demonstrated intense focal uptake within the tumour (maximum standardized uptake value = 10.5) (Figure 3). The scan did not show any abnormality in other regions. Surgical excision of the tumour was performed. Histological examination of the sections revealed proliferation of oval-to-short spindle-shaped cells arranged in sheets or a haphazard pattern, accompanied by fibrocollagenous stroma, abundant various-sized vessels, microcysts and thickened anastomosed bone trabeculae. Immunohistochemically, the tumour cells were focally positive for FGF23 (not shown). The final diagnosis of PMT was confirmed in conjunction with the serological elevation of FGF23. The postoperative course was uneventful. The patient experienced a significant decrease in systemic bone pain and the laboratory data normalized immediately.

## Discussion

Clinical symptoms dramatically improve after removal of PMTs, and surgical excision is the first choice of treatment.^3^ Although pain relief can be obtained using radiofrequency ablation, symptomatic treatment alone is usually chosen for unresectable cases. The duration of symptoms varies from 9 months to over 20 years^1^ and is an important factor for differentiating between benign and malignant PMTs.^4^ These facts suggest that diagnostic imaging plays an important role in early detection of the tumour and subsequent treatment. Venous sampling for analysis of FGF23 in the whole body is sometimes helpful for localization of the responsible tumour,^5^ although localization was not achieved with this method in the present case.

PMT is a rare tumour that is associated with oncogenic osteomalacia.^1^ These tumours overexpress FGF23, which inhibits reabsorption of phosphate in the renal tubules and decreases 1,25-dihydroxy vitamin D levels. Subsequently, hypophosphataemia and osteomalacia occur.

Primary PMT frequently occurs in the soft tissues of the extremities, but rarely occurs in the spine. To our knowledge, only nine cases have been reported in the literature.^6–8^ The patients were 3 males and 6 females, ranging in age from 14 to 66 years (mean 48.0 years). The tumours were located in the cervical (3 cases), thoracic (3 cases), lumbar (1 case) and sacral (2 cases) spines. PMT often appears as a low density on non-enhanced CT images,^6^ and sclerosis is sometimes seen, which was consistent with our case. Microcystic changes may be present in some PMT cases,^7,8^ but they are not pathognomonic. Previous reports have shown that the differences between benign and malignant PMT are found in the infiltrative pattern of the primary tumour and the tumour size.^4^ Nakanishi et al^9^ reported that whole-body MRI using a single-shot short *T*_1_ recovery-echo planar imaging sequence could show PMT as high intensity. These results imply that CT scan and MRI might be useful for assessing the localization of PMT. However, these imaging findings are non-specific and PMTs could be missed if they are small.

^68^Ga-DOTATOC imaging focuses on detecting neuroendocrine tumours and has some beneficial pharmacokinetic properties. ^68^Ga-DOTATOC PET/CT scan has higher spatial resolution and has been reported to better detect neuroendocrine tumours compared with single-photon emission CT.^2^ In addition, a ^68^Ga-DOTA-labelled somatostatin analogue is superior to ^18^F-fludeoxyglucose, which is widely used clinically for tumour imaging, as well as for the detection of neuroendocrine tumours. Histologically, most PMTs are composed of small, bland, spindled-shaped cells that produce a distinctive smudgy matrix, with a well-developed capillary network.^1^ The identification of FGF23 production by tumour cells has been used to confirm the diagnosis of PMT.^1,10^

A subset of cases also show a prominent fibrohistiocytic reaction and woven bone production.^1^ In the present case, CT scan showed a low-density tumour with a well-defined sclerotic margin. On MRI, the tumour was hypointense on *T*_2_ weighted imaging and showed heterogeneous enhancement. ^68^Ga-DOTATOC PET/CT scan demonstrated intense focal uptake within the tumour. The hypointensity on *T*_2_ weighted imaging may be due to the histological fibrocollagenous stroma and the focal uptake may correspond to excess FGF23 production. We found no reports that compared imaging and histological findings of PMT of the spine.

In conclusion, ^68^Ga-DOTATOC PET/CT scan for somatostatin receptor imaging is much better for the localization of PMT compared with CT scan or MRI. CT scan and MRI are useful for assessing the extent of the tumour.

## Learning points

1. Detection of PMT can be challenging, but the clinical symptoms dramatically improve after removal of the tumour.
2. ^68^Ga-DOTATOC PET/CT scan for somatostatin receptor imaging is useful in the localization of PMT.
3. CT scan and MRI should be modalities of choice in evaluating the extent of the tumour.

## Consent

Written informed consent was obtained.
